# β-Carboline Compounds, Including Harmine, Inhibit DYRK1A and Tau Phosphorylation at Multiple Alzheimer's Disease-Related Sites

**DOI:** 10.1371/journal.pone.0019264

**Published:** 2011-05-06

**Authors:** Danielle Frost, Bessie Meechoovet, Tong Wang, Stephen Gately, Marco Giorgetti, Irina Shcherbakova, Travis Dunckley

**Affiliations:** 1 Neurogenomics Division, Translational Genomics Research Institute, Phoenix, Arizona, United States of America; 2 Translational Drug Development, Translational Genomics Research Institute, Scottsdale, Arizona, United States of America; 3 MediProPharma, Inc., Salt Lake City, Utah, United States of America; 4 Arizona Alzheimer's Research Consortium, Phoenix, Arizona, United States of America; Alexander Flemming Biomedical Sciences Research Center, Greece

## Abstract

Harmine, a β-carboline alkaloid, is a high affinity inhibitor of the dual specificity tyrosine phosphorylation regulated kinase 1A (DYRK1A) protein. The DYRK1A gene is located within the Down Syndrome Critical Region (DSCR) on chromosome 21. We and others have implicated DYRK1A in the phosphorylation of tau protein on multiple sites associated with tau pathology in Down Syndrome and in Alzheimer's disease (AD). Pharmacological inhibition of this kinase may provide an opportunity to intervene therapeutically to alter the onset or progression of tau pathology in AD. Here we test the ability of harmine, and numerous additional β-carboline compounds, to inhibit the DYRK1A dependent phosphorylation of tau protein on serine 396, serine 262/serine 356 (12E8 epitope), and threonine 231 in cell culture assays and *in vitro* phosphorylation assays. Results demonstrate that the β-carboline compounds (1) potently reduce the expression of all three phosphorylated forms of tau protein, and (2) inhibit the DYRK1A catalyzed direct phosphorylation of tau protein on serine 396. By assaying several β-carboline compounds, we define certain chemical groups that modulate the affinity of this class of compounds for inhibition of tau phosphorylation.

## Introduction

The dual-specificity tyrosine phosphorylation regulated kinase 1A (DYRK1A) gene is located within the Down syndrome critical region on chromosome 21. Overexpression of DYRK1A has been proposed to be a significant contributor to the underlying neurodevelopmental abnormalities associated with Down syndrome. Transgenic animals overexpressing DYRK1A show marked cognitive deficits and impairment in hippocampal dependent memory tasks [1], [2]. Studies in cell culture models and transgenic models of Down syndrome that overexpress DYRK1A implicate the DYRK1A kinase in the generation of both amyloid and tau pathologies associated with the early onset Alzheimer's disease (AD) that is uniformly observed in Down Syndrome [3], [4], [5], [6]. We and others have shown that DYRK1A is important for phosphorylation of tau protein on multiple sites in several cellular models [3], [4], [6], [7]. Interestingly, DYRK1A protein has been found to be associated with neurofibrillary tangles (NFTs) in sporadic Alzheimer's disease [8] and some studies have found a genetic association between SNPs within the DYRK1A locus and Alzheimer's disease in some populations [3] but not others [9]. These combined observations raise the possibility that DYRK1A may be a critical contributor to tau dysfunction and tau pathology of Alzheimer's disease and, moreover, that this kinase may be an important therapeutic target for pharmacological interventions seeking to modify the course of tau pathology in AD.

The family of β-carboline alkaloids, characterized by a core indole structure and a pyridine ring, are naturally occurring compounds in some plant species that affect multiple central nervous system targets. These include the 5-hydroxytryptamine receptor substypes 5-HT_2_ and 5-HT_1A_ [10], the NMDA receptor [11], monoamine oxidase (MAO-A) [12], and dopaminergic signaling pathways [13], [14], [15]. In addition to these targets, the β-carboline alkaloid, harmine, has recently been reported to be a high affinity inhibitor of DYRK1A kinase activity [16], [17], suggesting that harmine, and possibly other β-carboline derivatives, could alter tau phosphorylation.

In this study, we extend previous published findings from our lab and others that show DYRK1A is involved in phosphorylation of tau protein on sites that are hyperphosphorylated during the course of tau pathology in AD and show that certain β-carboline alkaloids can significantly reduce the levels of phosphorylated tau protein. Specifically, we identify DYRK1A dependent tau phosphorylation on threonine 231 and serine 396. We further show that harmine and other β-carboline compounds inhibit DYRK1A dependent tau phosphorylation with varying affinities that are dependent upon several structural features of the molecules. These results suggest that this class of compounds warrant further investigation as candidate tau-based therapeutics to alter the onset or progression of tau dysfunction and pathology in Alzheimer's disease and other tauopathies.

## Methods

### siRNA transfection

4R0N tau overexpressing H4 neuroglioma cells [7] were maintained in Dulbecco's Modified Eagle Medium (Invitrogen) supplemented with 10% fetal bovine serum (Invitrogen), 1% penicillin-streptomycin, geneticin (0.25 mg/ml), and 2 mM L-Glutamine (Invitrogen). Cells were maintained by splitting 1∶10 at 90% confluency. Prior to any experimentation, cells were 70–75% confluent to ensure cells were in their active growth phase. To test effects of DYRK1A knockdown on tau phosphorylation, cells were transfected with DYRK1A siRNA. Prior to treating cells with DYRK1A siRNA, siRNA was first complexed with siLentfect lipid transfection reagent (Bio-Rad) and reduced serum medium (Invitrogen) using a 6 well plate format. The final effective siRNA molarity used was 22.85 nM per well. Cells were grown for 96 hours at 37°C, 5% CO_2_. Cell lysates were prepared using the Complete Lysis-M, EDTA-free kit (Roche Applied Science) and total protein concentration was quantified using the BCA protein assay (Pierce). Westerns for the multiple forms of tau were performed as described below.

### Western Blotting

For all cell-based experiments, including siRNA treatments and compound treatments, cells were treated for 96 hours at 37°C, with 5% CO_2_. Cell lysates were then prepared using the Complete Lysis-M, EDTA-free kit (Roche Applied Science) supplemented with phosphatase inhibitor cocktails 1 and 2 (Sigma). Lysates were quantified using the BCA protein assay (Pierce). Protein from lysates (30 µg total protein per lane) was separated on SDS-PAGE gels and transferred to nitrocellulose membrane. Membranes were blocked in 5% blocking solution for one hour at room temperature (RT). Blocking buffer solution used for detection of non phosphorylated protein contained 5% Non-fat dry milk in 1×-TBS-T (50 mM Tris-HCl pH 7.4, 137 mM NaCl_2_, 2.7 mM KCl, 0.1% Tween). For detection of phosphorylated protein, blocking buffer solution contained 5% Bovine Serum Albumin in 1× TBS-T. Membranes were probed with primary antibody (various dilutions depending on the epitope – see below) in blocking buffer overnight at 4°C on a rocker. Membranes were subsequently washed with 1× TBS-T and probed with secondary antibody in blocking buffer for forty-five minutes using a 1∶25,000 dilution of HRP-GAM or HRP-GAR (Jackson Immunoresearch), depending on the species (mouse or rabbit) in which the primary antibodies were raised. Following incubation with secondary antibody, membranes were washed in 1× TBS-T and developed with Super Signal West Femto Maximum Sensitivity Substrate Kit (Promega) and digitally imaged. Protein band signal saturation was assessed before any further analysis of multiple forms of tau. Alpha Innotech Fluoro Chem SF imaging software verifies degrees of saturation when signal intensity is beyond the dynamic range (which is from 0–65,535). Protein band signal intensities used for quantification were within the instrument's dynamic range.

To test multiple primary antibodies, membranes were stripped for 15 minutes at RT using ReBlot Plus Mild Antibody Stripping Solution (Millipore). Membranes were then washed for 5 minutes in 1× TBS-T and blocked for one hour in 5% blocking solution at RT. For verification of protein loading, membranes were reprobed overnight at 4°C with an anti-Tubulin primary antibody (1∶1000 dilution; Cell Signaling). Primary antibodies used for detection included anti-tau (1∶2000 dilution; Dako), 12E8 tau (1∶7500 dilution; Peter Seubert, Elan Pharmaceuticals), pT231 tau (1∶1000 dilution; Abcam), pS396 tau (1∶5000 dilution; Abcam), and anti-DYRK1A (1∶500 dilution; Santa Cruz).

### Compound treatments

Cells undergoing any treatment, including β-carboline derivative dosing and siRNA treatment, were maintained in Dulbecco's Modified Eagle Medium (Invitrogen) supplemented with 10% fetal bovine serum (Invitrogen) and 2 mM L-Glutamine (Invitrogen). Viability assays were performed using a 96-well pate format. Metabolic activity was measured 12 hours after the addition of 10% alamar Blue (Invitrogen) directly to attached cells in full medium. This assay was based on the ability of metabolically active cells to convert alamar Blue reagent into a fluorescent signal proportional to innate metabolic activity. Once the ideal IC_50_ value for viability was identified, effects on multiple forms of tau were investigated after treating with the β-carbolines indicated in Table S1 using the larger 6 well plate format. For both the viability assay and cell culture tau assays, cell culture media was removed and cells were treated with freshly made drug in new media every 24 hours for four days. For cell culture tau assays, protein lysates were prepared after 96 hours of treatment. All compounds were solubilized in dimethylsulfoxide (DMSO), diluted in growth medium to their respective 0.01 µM, 0.1 µM, 1 µM and 10 µM final working dilutions and added directly to cultured cells. The final DMSO percentage in culture for all compounds and concentrations tested was 0.1%. All treatments conditions were compared to their respective controls which contained DMSO at 0.1% in growth medium.

### *In Vitro* Kinase Assay

Evaluation of DYRK1A kinase activity was determine by incubating 0.08 ug of recombinant human DYRK1A protein (Invitrogen) with 0.15 ug of 4R2N recombinant human Tau (SignalChem) in 1× kinase buffer (25 mM Tris-HCl (pH 7.5), 5 mM beta-glycerophosphate, 2 mM dithiothreitol (DTT), 0.1 mM Na3VO4, 10 mM MgCl2 - Cell Signal) and 1 mM ATP in a final volume of 20 ul for 30 minutes @ 30C. For testing the effects of the β-carboline derivatives, recombinant human DYRK1A was pretreated with compounds for 10 minutes prior to the addition of kinase buffer, ATP, and recombinant human tau. The reaction was inactivated upon addition of 1× Novex LDS sample buffer and Novex sample reducing reagent, 50 mM DTT, (Invitrogen) followed immediately by heating for 10 minutes @ 95C. Phosphorylated Tau was resolved using 7% Tris Acetate gels and detected by western analysis. Westerns were probed for Phospho-Tau S396 (abcam) @ 1∶5,000 dilution and a secondary of Goat anti – Rabbit HRP (Jackson Immuno Labs) @ 1∶50,000 in 5% BSA. Membranes were stripped as above and reprobed with rabbit anti Human Total Tau (Dako) @ 1∶15,000 dilution and a secondary of Goat anti – Rabbit HRP (Jackson Immuno labs) @ 1∶100,000 dilution in 5% milk. For quantitation purposes, both bands of pS396 phosphorylated tau were used in the *in vitro* phosphorylation assays.

## Results

### Reduced DYRK1A expression affects tau phosphorylation at multiple sites

**Figure 1:**
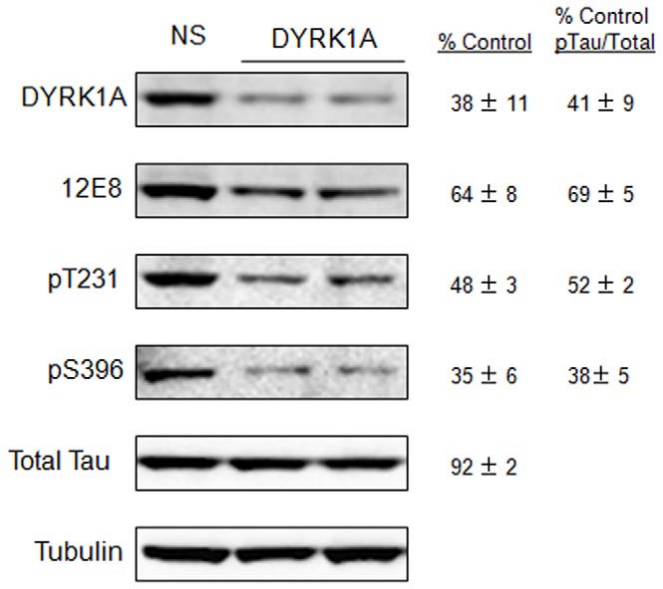
DYRK1A is required for efficient phosphorylation of pT231, pS396 and 12E8 tau. H4 neuroglioma cells overexpressing four repeat tau (4R0N) were transfected with siRNA targeting the DYRK1A transcript. Silencing of DYRK1A was confirmed with anti-DYRK1A antibody (top panel). Percent control values represent the average of three independent siRNA transfections and westerns. NS refers to the non-silencing control. 12E8 refers to the dual phosphorylation epitope pS262/pS356.

We previously reported that silencing of DYRK1A expression causes reduced tau phosphorylation at the 12E8 epitope (phosphorylated serines 262 and 356) [7]. Here we show that RNAi-mediated silencing of DYRK1A expression simultaneously affects multiple additional AD-relevant tau phosphorylation sites, including threonine 231 and serine 396 (Figure 1). We transfected H4 neuroglioma cells that overexpress 4R0N tau with siRNA specific for DYRK1A. Results showed that reduction of DYRK1A expression to 38% of control leads to pT231 and pS396 tau expression that is 48% and 35% of control nonsilencing siRNA, respectively. These results are consistent with the hypothesis that DYRK1A may be a promiscuous tau kinase and are also consistent with prior studies that have shown DYRK1A phosphorylation of tau on several other sites, including threonine 212 [3], [4], serine202, and serine 404 [6]. The finding that DYRK1A is involved in the phosphorylation of sites that control key microtubule binding functions of tau (S262, T231), as well as sites that are phosphorylated relatively late during the formation of neurofibrillary tangles (S396, S404), raises the interesting possibility that DYRK1A could be an important site of regulatory control for tau function and for the formation of tau pathology during the progression of tauopathies.

### The high affinity DYRK1A inhibitor, harmine, affects tau phosphorylation on multiple sites

**Figure 2:**
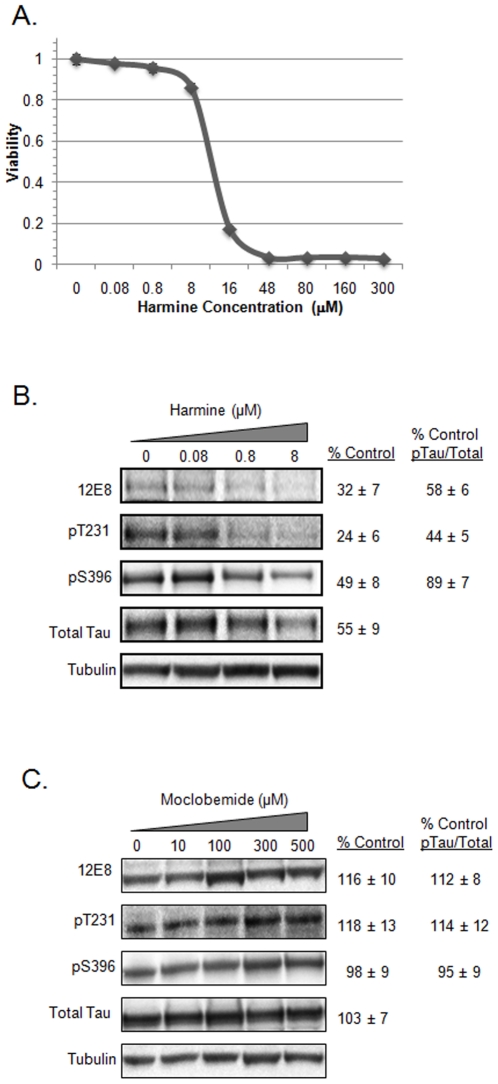
Harmine, a DYRK1A antagonist, inhibits tau phosphorylation. Shown in (A) is the toxicity profile of harmine against the H4 neuroglioma cell line. The IC_50_ for viability was 12 µM. In (B), are results of a dose-response treatment of H4-tau cells with harmine at the indicated concentrations. Reductions to total tau and all three phosphorylated forms of tau tested were observed. % control values represent the amount of the respective tau forms present following treatment with 8 µM harmine, although 0.8 µM harmine also reduced phospho-tau levels significantly. Harmine treatment was performed as described in the Methods. For (C), results for moclobemide, an MAO-A selective antagonist, are shown. % control values represent the amount of each form of tau present following treatment with the highest 500 µM concentration.

Harmine, a naturally occurring β-carboline alkaloid, is a potent inhibitor of DYRK1A with a reported IC_50_ of ∼80 nM in an *in vitro* kinase assay using synthetic peptide substrate [17]. Based on our findings that DYRK1A silencing reduces multiple phosphorylated tau species, we tested harmine for effects on tau phosphorylation in the H4 neuroglioma cell line. We first determined the toxicity profile for harmine (Figure 2A). Results of increasing concentrations of harmine showed that 12 µM resulted in 50% cell viability. Based on this toxicity profile and the reported *in vitro* IC_50_ value for harmine against DYRK1A, we selected doses of 80 nM, 800 nM and 8 µM for the tau phosphorylation assays. Harmine reduced the expression of each phospho-tau species tested, including 12E8 (pS262/pS356), pT231, and pS396 (Figure 2B). Significant (P<0.05) reductions to 12E8 and pT231 tau were noted at 0.8 µM and 8 µM concentrations. However, it is important to note that harmine at 0.8 µM and 8 µM also reduced the levels of total tau protein consistent with the reductions detected with the various phospho-tau antibodies. Even accounting for reductions to total tau levels, significant reductions to 12E8 (58% of control) and pT231 (44% of control) levels remained (Figure 2B). pS396 effects are largely accounted for by overall reductions to total tau levels.

In addition to DYRK1A inhibition, harmine has been reported to be a selective inhibitor of monoamine oxidase (MAO-A) [12]. To test if effects on tau could result from MAO-A inhibition, we tested another MAO-A selective antagonist, moclobemide, for effects on tau. Moclobemide has a reported IC_50_ against MAO-A of 3.9 µM [18]. Results showed that this MAO-A antagonist did not reduce levels of either total tau or of specific phosphorylated forms of tau protein at doses up to 500 µM (Figure 2C). These results suggest that the effects of harmine on tau do not result from MAO-A inhibition.

### Additional β-carboline alkaloid derivatives alter the expression of multiple tau species

**Figure 3:**
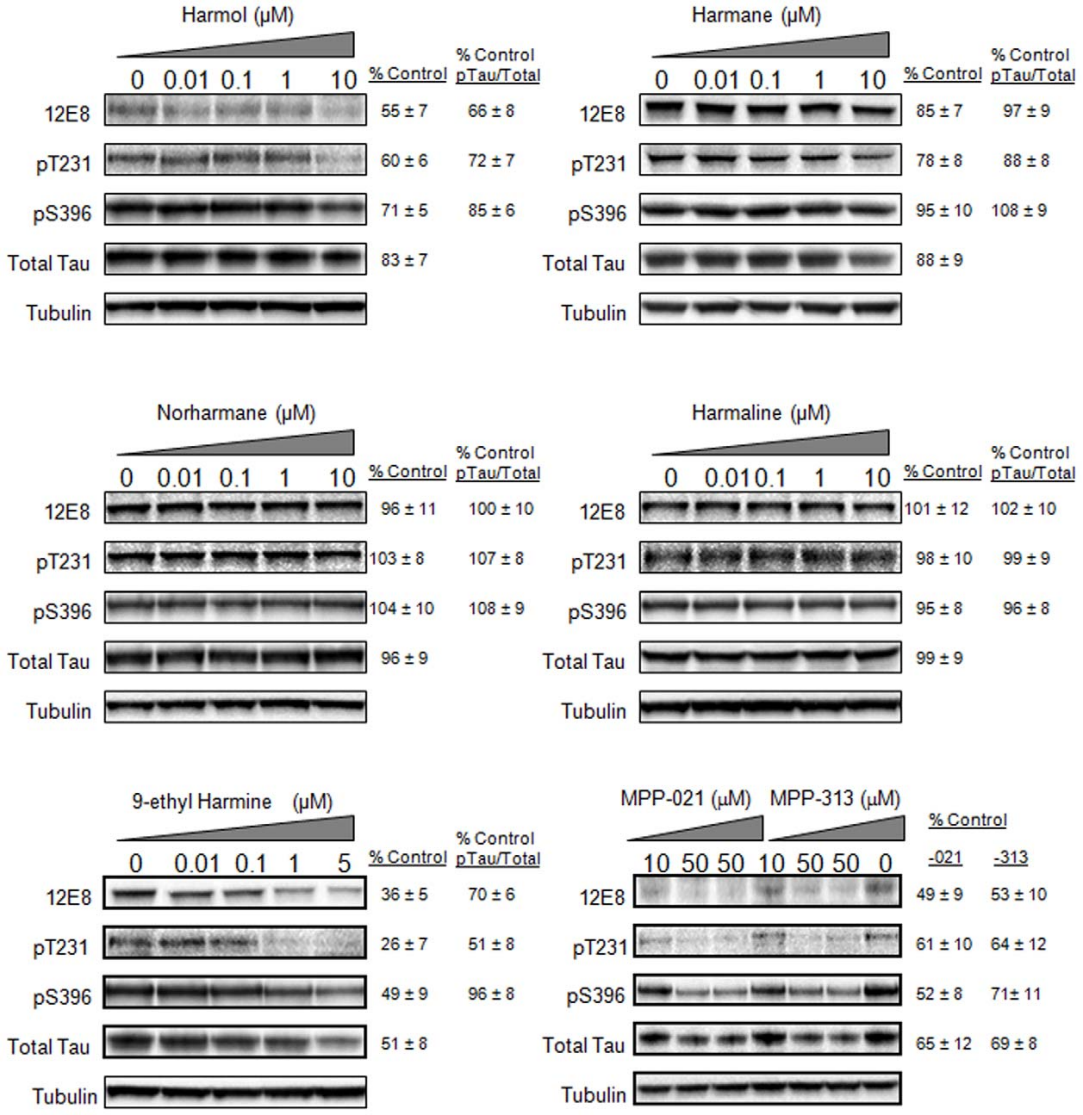
Multiple β-carboline derivatives affect levels of total tau and phosphorylated tau. Each indicated compound was tested at the concentrations shown below each compound name. % control values represent the effect seen at the highest concentration tested for each compound. 9-ethylharmine clearly showed the most potent effect in this assay, significantly reducing total and phospho tau levels at a 1 µM concentration.

**Figure 4:**
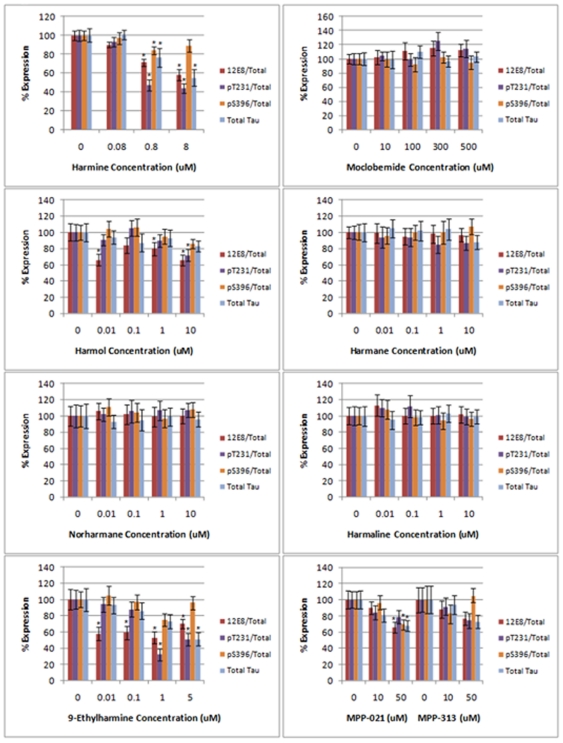
Quantification of the inhibition of tau phosphorylation by multiple β-carbolines. Quantification of tau phosphorylation data from the H4 cells is shown for each compound tested. The phospho-tau data have been normalized to account for any changes to total tau levels. Effects on total tau are indicated in each graph. Significance at p<0.05, as assessed by Student's T-test, is indicated by asterices above the error bars on the graphs. Error bars (standard deviation) from three independent replicates are shown.

Based on results for harmine, we tested additional β-carboline derivatives, including harmol, harmane, harmaline, norharmane, 9-ethylharmine, and two novel proprietary compounds MPP-021 and MPP-313 (MediProPharma, Inc.: patents pending). We first performed toxicity assays for each compound in our H4 cell line (Table S1). We then tested each compound for effects on phospho-tau and total tau expression (Figure 3). Quantification of phosphorylated tau levels is shown in Figure 4. Data for phosphorylated tau levels have been corrected for effects of the compounds on total tau levels. Quantification of absolute phospho-tau levels is included as supplementary material (Figure S1). There was a positive correlation between the toxicity of each compound and the sensitivity with which each compound reduced total tau levels and the levels of phosphorylated forms of tau. For toxicity, the rank order of the compounds was 9-ethylharmine>harmine>harmol>harmane>harmaline>MPP-021>norharmane>MPP-313. In terms of the sensitivity with which each compound reduced tau levels, only 9-ethylharmine, harmine, and harmol showed significant effects (p<0.05) in reducing total tau and phosphorylated tau levels at doses ≥1 µM. As with harmine results in Figure 2, reductions to 12E8 tau and pT231 tau remained significant after accounting for total tau reductions (Figure 4). MPP-021 and MPP-313 significantly reduced (p<0.05) tau levels at 50 µM. The 9-ethylharmine and harmine compound treatments showed significant reductions at 1 µM and 0.8 µM, respectively. These lower doses have no detectable effect on the viability of the cells. We therefore conclude that reducing tau levels beyond ∼50% of the control levels, as occurs at higher concentrations, leads to significant cellular toxicity rather than the alternative of the observed reductions in tau resulting from general drug-induced toxicity.

### Harmine and other β-carboline compounds inhibit the direct phosphorylation of tau by DYRK1A

**Figure 5:**
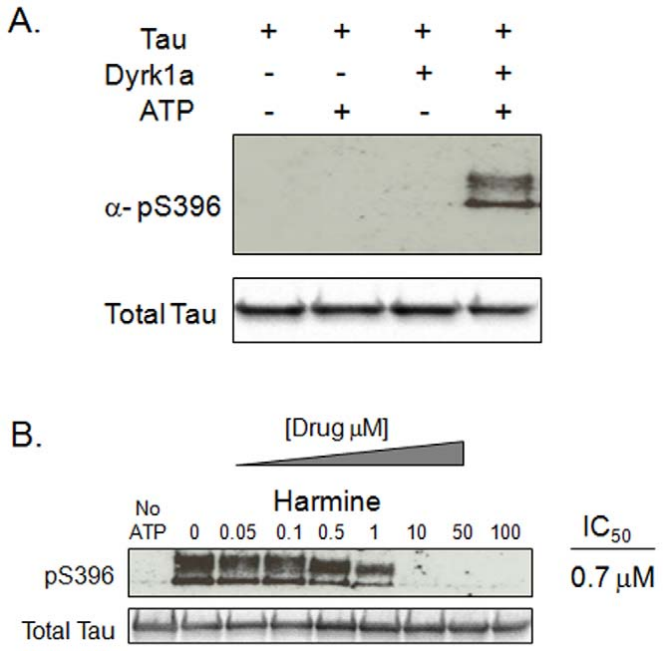
Harmine inhibits the DYRK1A catalyzed direct phosphorylation of tau protein on serine 396. Shown in (A) are results of an *in vitro* phosphorylation assay utilizing recombinant DYRK1A and tau proteins. A doublet pS396 tau phosphorylation is observed only in the presence of tau, DYRK1A, and ATP. In (B), harmine potently inhibits the direct phosphorylation of tau protein by DYRK1A with an IC_50_ of 0.7 µM.

DYRK1A has been reported to directly phosphorylate tau protein on T212, S202, and S404 [3], [4], [6]. To determine if the effects observed with various β-carboline derivatives above could result from the inhibition of direct phosphorylation of tau protein by DYRK1A, we first tested if DYRK1A could directly phosphorylate serine 396. Using an *in vitro* phosphorylation assay with recombinant DYRK1A and tau proteins, we confirmed that DYRK1A could directly phosphorylate tau protein (Figure 5A). Phosphorylation occurred only in the presence of tau protein, DYRK1A protein, and ATP. We observed a doublet of pS396 phosphorylated tau. Because the primary protein band recognized by the total tau antibody is the molecular weight of the lower band of this pS396 doublet, the doublet is unlikely to result from any protein degradation. Rather, it may be that the higher molecular weight band contains phosphorylations in addition to serine 396. This explanation is consistent with reported literature indicating a role for DYRK1A in the phosphorylation of tau protein on S202, S404, and T212 [3], [4], [6]. The observed pS396 tau phosphorylation was potently inhibited by harmine with an IC_50_ of 0.7 µM (Figure 5B).

**Figure 6:**
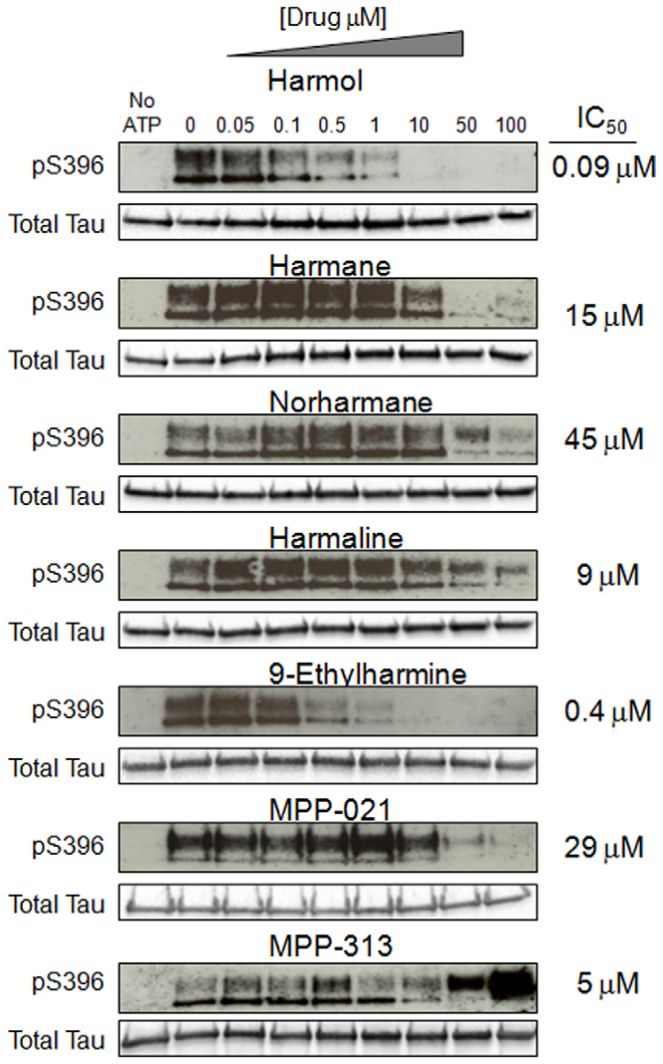
Structurally distinct β-carboline derivatives inhibit DYRK1A-dependent pS396 tau phosphorylation with varying affinities. Shown are *in vitro* phosphorylation results for all compounds in this study. The compounds tested are indicated above the respective western results for each compound. The concentrations (in µM) are indicated at the top of the first panel and are the same for each compound tested. The IC_50_ values calculated from these assays are indicated in the right column, next to the western results for each compound.

**Figure 7:**
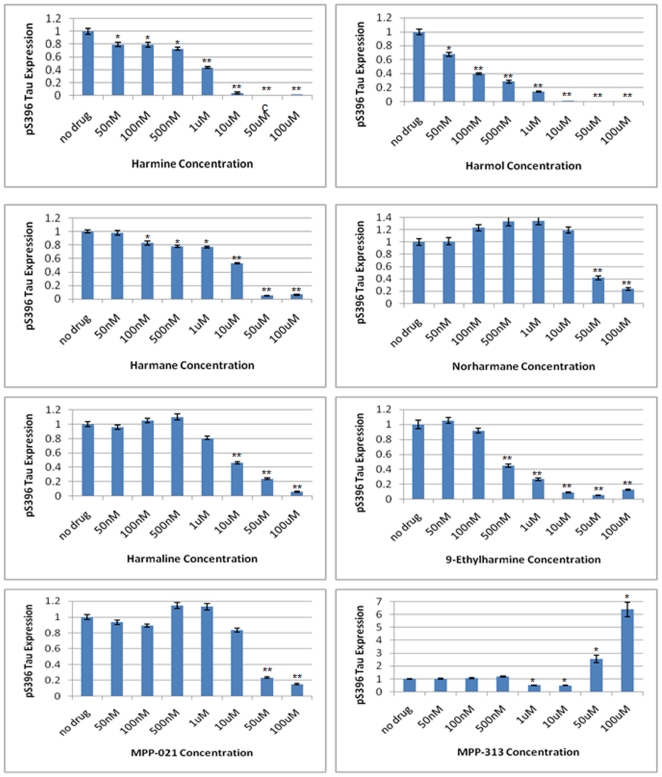
Quantification of β-carboline affinities for inhibition of pS396 phosphorylation *in vitro*. Quantification of the *in vitro* phosphorylation data at each drug concentration tested is shown. Error bars represent the standard deviation of three independent replicates. Significance at p<0.01, as assessed by Student's T-test, is indicated by a single asterix above the error bars on the graphs. Significance at p<0.001 is indicated by two asterices above the error bars.

We next tested each β-carboline derivative compound in this *in vitro* assay (Figure 6 and Figure 7). Several interesting observations emerged from this series of studies. We first determined the IC_50_ values for each compound for the inhibition of DYRK1A dependent tau phosphorylation at serine 396. These results reflect the rank ordered affinities for each compound that were obtained in the cell based tau phosphorylation assays and the toxicity assays (Table S1 and Figures 2B and Figure 3), with one exception. Harmol was the most potent inhibitor in this *in vitro* phosphorylation assay with an IC_50_ of 90 nM, followed by 9-ethylharmine (400 nM) and harmine (700 nM). Harmol was the third ranked compound in both the toxicity and cell-based tau assay. Reasons for this slight disconnect are unclear, but could be related to differential cellular metabolism of the free hydroxyl group on carbon 7 of harmol.

The addition of an ethyl group to N-9 of harmine reduced the IC_50_ nearly 2-fold, suggesting that additional modifications on this nitrogen might increase the affinity of harmine for DYRK1A more substantially. Harmane, norharmane, and harmaline were more than an order of magnitude lower affinity than harmine, consistent with the relatively muted effects of these compounds in our cell-based tau assay (Figure 3).

The MPP-313 compound reduced phosphorylation of the lower molecular weight form of phosphorylated tau protein with an IC_50_ of 5 µM. However, effects on the larger molecular weight band were striking. Levels of this larger phosphorylated tau protein decline to near 50% of the no drug treated sample at a concentration of 5 µM, but then sharply reverse and increase significantly beyond the no drug treated control at doses of 50 µM (255% control) and 100 µM (640% control) (Figure 6). This was the only β-carboline derivative tested that displayed this pattern. All other compounds showed consistent effects on both molecular weight forms of pS396 tau. While we cannot yet explain this observation, it does suggest that at higher concentrations MPP-313 induces DYRK1A activity through a yet-to-be determined mechanism.

## Discussion

### DYRK1A is involved in phosphorylation of multiple sites on tau

Previous literature indicates that DYRK1A can phosphorylate tau protein on T212, S202, S404, and the 12E8 epitope (S262/S356) [3], [4], [6], [7]. Here we provide evidence that DYRK1A is also involved in tau phosphorylation at threonine 231 and serine 396 (Figure 1). This growing list of phosphorylation sites affected by DYRK1A, which now includes key sites regulating microtubule affinity (T231, S262) [19], [20] and references therein], tau toxicity (T231, S262, T212) [21], and sites hyperphosphorylated coincident to NFT pathology (S396, S404) [22] suggest that DYRK1A could be a critical regulator of tau function and dysfunction during the course of AD. As such, targeting this kinase pharmacologically may provide a means to modify the course of tau dysfunction and pathology in AD and other tauopathies.

### DYRK1A directly phosphorylates tau on serine 396

DYRK1A can directly phosphorylate tau protein on serine 396 (Figure 4A). Due to high baseline phosphorylation of tau on threonine 231 and on the 12E8 epitope in these preparations of recombinant tau (data not shown), we were unable to test for direct tau phosphorylation by DYRK1A on these epitopes. However, our data are consistent with these sites either being directly phosphorylated by DYRK1A or being controlled by a pathway that is dependent on DYRK1A activity. Whether these pathways ultimately result in the direct phosphorylation of tau on T231 or the 12E8 epitope or whether these sites are ultimately affected through indirect mechanisms, such as altered tau protein half-life resulting from decreased tau phosphorylation at other sites, remains to be determined.

### Harmine, a DYRK1A inhibitor, alters the expression of multiple forms of phosphorylated tau

Based on our RNA interference data implicating DYRK1A in the phosphorylation of tau protein at multiple sites (Figure 1), we tested the high affinity DYRK1A inhibitor harmine for effects on tau. Harmine showed a clear dose response profile for the inhibition of tau phosphorylation (Figure 2B) at doses that elicited no toxicity (Figure 2A). The increased toxicity that occurred at higher concentrations prevented obtaining an IC_50_ for tau effects in our cell-based tau assay. However, because harmine significantly affected tau levels prior to observed toxicity, we conclude that the toxicity seen at higher concentrations beyond 8 µM likely results from excessive reductions to tau protein levels. This interpretation is consistent with our prior published findings that reductions to overall tau levels via siRNA knockdown of the tau transcript, and concomitant decreases to levels of phosphorylated tau, lead to significant cellular toxicity [7].

Although harmine significantly reduced total tau levels, reductions to both 12E8 and pT231 phosphorylated tau remained highly significant (Figure 4). However, interestingly, the reductions to pS396 tau at higher concentrations mirror the reductions to total tau levels. This is a somewhat striking observation since DYRK1A can clearly directly phosphorylate tau protein on serine 396 (Figure 5) and suggests at least two possibilities. First, phosphorylation of serine 396 could be very tightly correlated to the overall stability of tau protein. Second, harmine and the other β-carbolines, all of which show the same differential effect on the relationship between pS396 tau and total tau (Figure 4), could target additional cellular proteins that then lead to this S396/Total Tau specific correlation. Further experiments will be needed to account for the relationship between these compounds, pS396 tau, total tau levels, and cellular toxicity.

### β-carboline alkaloid derivatives alter the levels of phosphorylated tau

Based on positive results for harmine, we tested several harmine derivatives, including fully aromatic β-carboline compounds (harmol, harmane, norharmane, 9-ethylharmine, and 3,4-substituted derivatives MPP-021 and MPP-313) and a dihydro-derivative (harmaline). Modifications to certain structural components of the β-carboline ring structure significantly affected the ability of these compounds to inhibit tau phosphorylation. A comparison of results for harmaline and harmine indicate that a fully aromatic ring structure provides higher affinity for tau inhibition and toxicity (see Table S1, Figure 2B, Figure 3, Figure 6). Certain modifications to carbon 7 increase toxicity and tau effects (compare harmine and harmol to harmane). Also, the methyl group on carbon 1 appears to be important for the observed tau effects and toxicity (compare norharmane to harmane). Lastly, the addition of an ethyl group to N-9 increased the effects of harmine on tau and increased toxicity (compare 9-ethylharmine to harmine). The combination of results provide insights into which structural features of harmine could be targeted and altered to improve the tau effects.

Another important observation from these compound derivative studies was that correlations between tau reductions and cellular toxicity that were first observed with harmine, were also found with all of the β-carbolines tested. While we were initially hopeful that certain derivatives could separate the tau effects from the toxic effects, no compounds separated these effects. This may well result from a causative relationship between excessive tau reductions and toxicity, consistent with our prior results targeting tau expression with siRNA [7]. This may have implications in the development of therapeutic strategies designed against tau expression or phosphorylation.

### Conclusions

Pharmacologic inhibition of tau phosphorylation at certain key sites that regulate the functional activity of tau or that promote the aggregation of tau in to neurofibrillary tangles may provide a promising approach for the treatment of AD and other tauopathies. We show that the β-carboline alkaloids inhibit DYRK1A kinase activity and reduce the levels of multiple phosphorylated forms of tau protein that are important in the pathological progression of AD. Further refinement of this class of compounds on functional groups that are important determinants of their affinity for DYRK1A could lead to high affinity inhibitors of tau phosphorylation.

## Supporting Information

Figure S1 Quantification of the inhibition of tau phosphorylation by multiple β-carbolines. Quantification of the absolute tau phosphorylation data from the H4 cells is shown for each compound tested. Data have not been normalized to account for changes to total tau levels. Significance at p<0.05, as assessed by Student's T-test, is indicated by asterices above the error bars on the graphs. Error bars (standard deviation) from three independent replicates are shown.
(TIF)

Table S1 β-carboline compounds tested in this study. Shown in columns from left to right are the compound names, chemical structures, concentration resulting in 50% viability in the H4 neuroglioma cell line used in all of the cell-based tau assays.
(TIF)
