# Supplementary figures and images for: β-Carboline Compounds, Including Harmine, Inhibit DYRK1A and Tau Phosphorylation at Multiple Alzheimer's Disease-Related Sites
Source: PLoS One. 2011 May 6;6(5):e19264. doi: 10.1371/journal.pone.0019264 (PMC3089604; doi:10.1371/journal.pone.0019264)

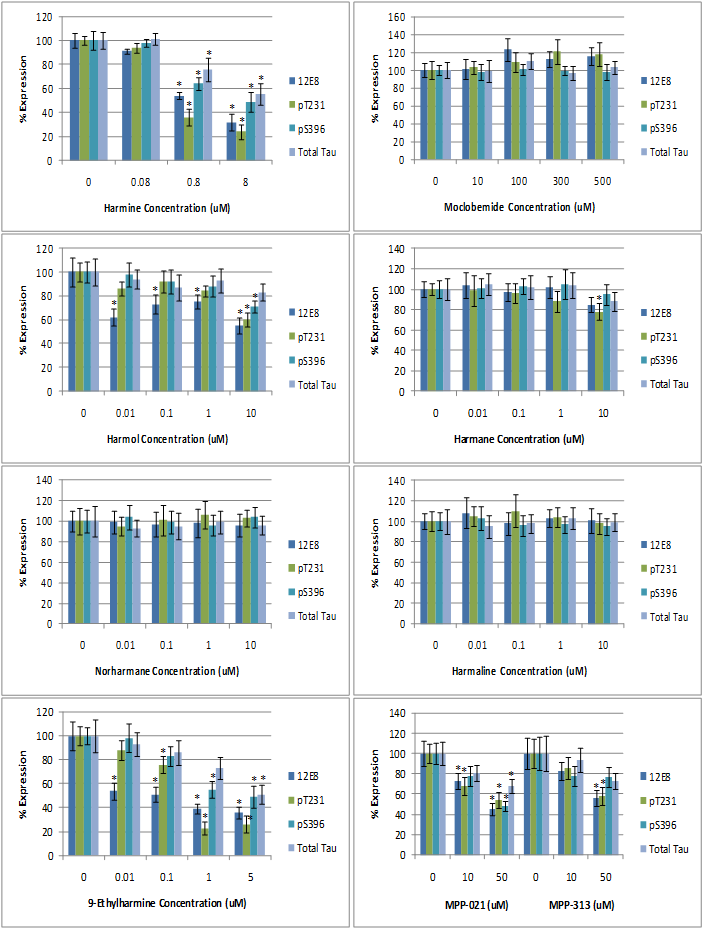

Supplement: Figure S1 — Quantification of the inhibition of tau phosphorylation by multiple β-carbolines. Quantification of the absolute tau phosphorylation data from the H4 cells is shown for each compound tested. Data have not been normalized to account for changes to total tau levels. Significance at p<0.05, as assessed by Student's T-test, is indicated by asterices above the error bars on the graphs. Error bars (standard deviation) from three independent replicates are shown. (TIF) [file pone.0019264.s001.tif]

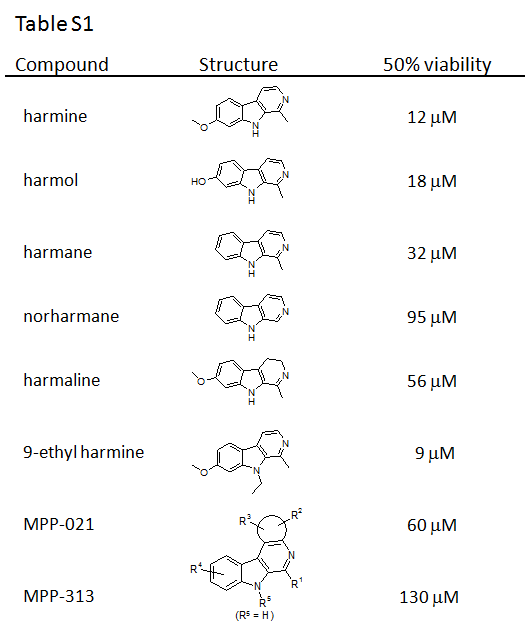

Supplement: Table S1 — β-carboline compounds tested in this study. Shown in columns from left to right are the compound names, chemical structures, concentration resulting in 50% viability in the H4 neuroglioma cell line used in all of the cell-based tau assays. (TIF) [file pone.0019264.s002.tif]
